# Outcomes of Acute Pancreatitis in Hospitalized Patients With Generalized Anxiety Disorder

**DOI:** 10.7759/cureus.43795

**Published:** 2023-08-20

**Authors:** Pooja Saiganesh, Alexander J Kaye, Shivani J Patel, Sarah R Meyers, Anna G Mathew, Weizheng Wang

**Affiliations:** 1 Internal Medicine, Rutgers University New Jersey Medical School, Newark, USA; 2 Psychiatry, Rutgers Robert Wood Johnson Medical School, Piscataway, USA; 3 Gastroenterology and Hepatology, Rutgers University New Jersey Medical School, Newark, USA

**Keywords:** deep vein thrombosis (dvt), inpatient mortality, sepsis, acute renal failure, generalized anxiety disorder, acute pancreatitis

## Abstract

Introduction

Acute pancreatitis (AP) is a common cause of hospitalization in the United States. There is evidence that chronic stress increases the risk for more severe AP episodes. One common form of chronic stress is generalized anxiety disorder (GAD). The purpose of this research was to investigate the impact of GAD on the outcomes of adult patients admitted to the hospital with AP.

Methods

Utilizing the 2014 National Inpatient Sample database and International Classification of Diseases, Ninth Edition Revision (ICD) codes, AP patients were selected. Common inpatient outcomes of AP patients with and without GAD were examined. The outcomes studied were acute renal failure, acute respiratory failure, sepsis, acute deep vein thrombosis, myocardial infarction, intestinal perforation, and inpatient mortality. A multivariate logistic regression analysis was conducted to assess if GAD was an independent predictor for these outcomes.

Results

Among 82,156 adult patients hospitalized for AP during the 2014 year, 10,611 of them had coexisting GAD. AP patients with comorbid GAD were found to have an increased likelihood of acute renal failure (aOR = 1.19, 95% confidence interval (CI) = 1.11-1.28, p < 0.001), sepsis (aOR = 1.09, 95% CI = 1.01 -1.19, p = 0.037), acute deep vein thrombosis (aOR = 1.63, 95% CI = 1.06-2.50, p = 0.025), and inpatient mortality (aOR = 1.62, 95% C = I 1.27-2.08, p < 0.001). There was no statistically significant difference found between the two cohorts for the outcomes of myocardial infarction and intestinal perforation.

Conclusion

In patients hospitalized with AP, those with coexisting GAD were found to have an increased risk of developing acute renal failure, sepsis, acute deep vein thrombosis, and inpatient mortality. There may be benefits to identifying AP patients with comorbid GAD at the time of admission and monitoring them more carefully during their hospitalization to help identify early signs of complications or prevent the negative outcomes seen in this study.

## Introduction

Acute pancreatitis (AP) is defined as an acute inflammation of the pancreas. It is most commonly caused by gallstones and alcohol, followed by other etiologies, such as infection, trauma, autoimmune disorders, drugs, or other obstructive processes [1]. AP is one of the most common gastrointestinal diagnoses that require hospital admission in the United States [2]. Hospitalization costs for AP are often more than $30,000 per hospitalization [3-5]. Over 200,000 adults per year are hospitalized due to AP, and the diagnosis of AP carries a mortality rate of around 1-2% [6,7].

The pathophysiology of AP involves premature activation of trypsinogen, leading to a cascade that activates zymogens, which results in damage to pancreatic tissue [1,8]. Classic symptoms include epigastric abdominal pain that may radiate to the back, nausea, and vomiting; these symptoms often coincide with elevations in serum amylase and lipase [7]. Severity is usually characterized by whether organ failure or systemic inflammatory response syndrome is present [1,2].

Prior research has suggested that chronic stress may increase the risk of more severe AP episodes as chronic stress is hypothesized to induce leukocyte infiltration and ischemia to the pancreatic parenchyma [1]. The resulting reactive oxygen species from ischemia can lead to increased levels of TNF-α production [1]. Increased TNF-α triggers several mechanisms that activate trypsinogen, disrupt actin cytoskeleton, and activate transcription factor NF-kB. These processes have all been found to sensitize the exocrine pancreas to injury [1].

One common source for chronic stress in the general population is generalized anxiety disorder (GAD) [9]. Found to occur in up to 20% of adults in a lifetime, GAD is a prevalent psychiatric disorder that presents as chronic, excessive worry across various environments that contributes to fatigue, restlessness, muscle tension, and sleep disturbance for at least six months [9,10]. The neurobiology associated with GAD has been linked to studies on a sustained threat response, which is associated with hormonal sequelae that induce cellular damage [9].

Although there has been an expanding body of research regarding the biological mechanisms of chronic stress, there has been limited data on the impact of GAD on hospitalized patients with AP. In this study, we explored the hospital-based outcomes of patients with AP who also have comorbid GAD to better understand the relationship between both disorders.

## Materials and methods

This retrospective cohort study was conducted to investigate adult patients (18 years old and older) who were hospitalized during the 2014 calendar year with AP. An institutional review board approval was not necessary for this research given that there was no patient-level data utilized. The National Inpatient Sample (NIS), a database created for the Healthcare Cost and Utilization Project, was the source for all the data collected in this study [11]. The NIS database is known for being the largest all-payer inpatient database in the United States [11]. The medical conditions and outcomes assessed in this research were identified from the NIS database using the International Classification of Diseases, Ninth Edition Revision, and Clinical Modification (ICD-9) codes. The data for every adult patient admitted with AP during the year 2014 were extracted from the NIS database. The data was then split between two groups: one group that had a diagnosis of comorbid GAD and the other group that did not have a comorbid GAD diagnosis. Between these two groups, the demographics and hospitalization data, including hospitalization cost, length of stay, race, sex, and age, were collected and then compared. The Charlson comorbidity index, a tool that can be used to adjust for numerous confounding variables, was also contrasted between the group of patients with comorbid GAD and the group of patients without comorbid GAD [12].

The Statistical Package for the Social Sciences (SPSS) version 28.0.0 (IBM Corporation, Armonk, NY) was used to conduct the statistical analyses. The clinical outcomes evaluated included acute renal failure, sepsis, acute respiratory failure, myocardial infarction, acute deep vein thrombosis, intestinal perforation, and inpatient mortality. Chi-squared tests and independent T-tests were utilized to compare the proportions and means, respectively, for the demographic data and the clinical outcomes data of the AP groups with and without GAD.

The statistical analyses were two-tailed and used a p-value of below 0.05 to delineate statistically significant data. Continuous variables were reported with means ± standard deviation (SD), and categorical variables were described utilizing percentages (%) and numbers (N). A multivariate logistic regression analysis was also performed to investigate whether GAD is a risk factor for the aforementioned outcomes, after adjusting for race, sex, age, cholelithiasis, hypertriglyceridemia, alcohol abuse, hypercalcemia, bile duct obstruction, and Charlson comorbidity index. The familywise error rate of the statistical analyses was not adjusted for given the increased risk of a type two statistical error that is associated with the correction, as well as the lack of a well-established benefit of using this type of correction [13].

## Results

For this study, 82,156 adult patients, hospitalized with AP during the 2014 year, were identified. Among these cases of AP, 10,611 of them were associated with comorbid GAD, while the other 71,545 cases did not have comorbid GAD. As observed in Table 1, demographic data and hospitalization characteristics significantly varied between the cohort with and without GAD. AP patients with GAD were on average younger (49.70 years old vs. 52.68 years old, p < 0.001), more likely to be female (59.2% vs. 47.4%, p < 0.001), and less likely to be Caucasian (79.7% vs. 84.4%, p < 0.001). Further, the GAD cohort had a smaller total hospitalization cost ($39,961 vs. $45,329, p < 0.001), as well as a lower Charlson comorbidity index (1.96 vs. 2.33, p < 0.001). No significant difference in length of stay (5.41 vs. 5.29, p = 0.096) was identified.

**Table 1:** Demographics, characteristics, length of stay, total hospitalization cost, and Charlson comorbidity index among acute pancreatitis patients with and without a history of generalized anxiety disorder.

	With generalized anxiety disorder	Without generalized anxiety disorder	p-value
N = 82,156	N = 10,611	N = 71,545	
Patient age, mean (SD)	49.70 (15.73)	52.68 (18.42)	<0.001
Sex, N (%)			<0.001
Female	6,279 (59.2%)	33,927 (47.4%)	
Male	4,328 (40.8%)	37,587 (52.6%)	
Race, N (%)			<0.001
White	8,004 (79.7%)	43,392 (84.4%)	
Black	903 (9.0%)	11,334 (16.7%)	
Hispanic	771 (7.7%)	8,928 (13.1%)	
Asian or Pacific Islander	73 (0.7%)	1,533 (2.3%)	
Native American	88 (0.9%)	662 (1.0%)	
Other	206 (2.1%)	2,120 (3.1%)	
Length of stay, in days (SD)	5.41 (6.62)	5.29 (6.97)	0.096
Total hospitalization cost, in $ (SD)	39,961 (59,172.57)	45,329 (88,393.97)	<0.001
Charlson comorbidity index (SD)	1.96 (2.03)	2.33 (2.27)	<0.001

As seen in Table 2, the clinical outcomes of patients admitted for AP varied significantly between the cohort with GAD and the cohort without GAD. Notably, patients with GAD had a decreased risk of acute renal failure (10.6% vs. 14.1%, p < 0.001), an increased risk of acute respiratory failure (3.7% vs. 3.2%, p = 0.011), a decreased risk of sepsis (6.9% vs. 8.2%, p < 0.001), a decreased risk of acute deep vein thrombosis (0.2% vs. 0.4%, p= 0.003), a decreased risk of myocardial infarction (0.6% vs. 0.9%, p = 0.011), and a decreased risk of inpatient mortality (0.7% vs. 1.4%, p < 0.001). No statistically significant difference in intestinal perforation (0.1% vs. 0.1%, p = 0.498) was observed between the two cohorts. These relationships are unadjusted for age, sex, race, cholelithiasis, hypertriglyceridemia, hypercalcemia, alcohol abuse, obstruction of the bile duct, and the Charlson comorbidity index. 

**Table 2:** Unadjusted clinical outcomes among acute pancreatitis patients with and without a history of comorbid generalized anxiety disorder.

Outcomes	With generalized anxiety disorder	Without generalized anxiety disorder	p-value
Acute renal failure	1,127 (10.6%)	10,059 (14.1%)	<0.001
Acute respiratory failure	2,663 (3.7%)	342 (3.2%)	0.011
Sepsis	731 (6.9%)	5,840 (8.2%)	<0.001
Acute deep vein thrombosis	26 (0.2%)	321 (0.4%)	0.003
Myocardial infarction	65 (0.6%)	663 (0.9%)	0.011
Intestinal perforation	11 (0.1%)	92 (0.1%)	0.498
Inpatient mortality	75 (0.7%)	1,019 (1.4%)	<0.001

In order to further investigate the impact of GAD on the previously mentioned outcomes, adjusted odds ratios (aORs) that adjust for differences in age, race, sex, bile duct obstruction, cholelithiasis, hypercalcemia, hypertriglyceridemia, alcohol abuse, and Charlson comorbidity index were subsequently calculated. As seen in Table 3, GAD was found to be an independent risk factor for acute renal failure (aOR = 1.19, 95% confidence interval (CI) = 1.11-1.28, p < 0.001), sepsis (aOR = 1.09, 95% CI = 1.01 -1.19, p = 0.037), acute deep vein thrombosis (aOR = 1.63, 95% CI = 1.06-2.50, p = 0.025), and inpatient mortality (aOR = 1.62, 95% CI = 1.27-2.08, p < 0.001). The p-values for the aORs of acute respiratory failure (aOR = 1.06, 95% CI = 0.94-1.20, p = 0.323), myocardial infarction (aOR = 1.21, 95% CI = 0.92-1.60, p = 0.166), and intestinal perforation (aOR = 0.96, 95% CI = 0.51-1.82, p = 0.907) did not meet the threshold of statistical significance. Tables 2-3 outline similar outcomes; however, the data appear to show conflicting findings. For example, in Table 2, there is a decreased incidence of inpatient mortality in the GAD cohort; however, Table 3 shows an increased odds ratio for inpatient mortality in the GAD group. This discrepancy can be linked to confounding factors that were adjusted for when calculating the aORs displayed in Table 3.

**Table 3:** Multivariate logistic regression analysis of the outcomes among acute pancreatitis patients. *Adjusted for age, sex, race, cholelithiasis, hypertriglyceridemia, hypercalcemia, alcohol abuse, obstruction of the bile duct, and the Charlson comorbidity index.

Outcomes	Adjusted odds ratio*	95% Confidence interval	p-value
Acute renal failure	1.19	1.11-1.28	<0.001
Acute respiratory failure	1.06	0.94-1.20	0.323
Sepsis	1.09	1.01-1.19	0.037
Acute deep vein thrombosis	1.63	1.06-2.50	0.025
Myocardial infarction	1.21	0.92-1.60	0.166
Intestinal perforation	0.96	0.51-1.82	0.907
Inpatient mortality	1.62	1.27-2.08	<0.001

## Discussion

Prior research reports have suggested an association between chronic physiologic stress and more severe presentations of AP [1]. Despite this association, the impact that anxiety had on AP remained unclear. To the best of our knowledge, this study is the first to evaluate the impact of GAD on the hospital-based outcomes of AP. 

This study found GAD to be a risk factor for acute renal failure in the setting of AP. Cytokines, such as TNF-α, IL-6, and TGF, that are released from neutrophilic granulocyte activation in AP progressively increase the risk of renal injury [14]. Studies have found that GAD is also associated with an increase in pro-inflammatory cytokines, such as TNF-α and IFN-γ, and a decrease in anti-inflammatory cytokines, such as IL-4 and IL-10 [15]. Elevated cytokine production in GAD could further exacerbate the TNF-α-induced ischemia and necrosis of the renal tubules and glomeruli that occurs in AP [14]. Increased TNF-α from GAD can also contribute to a rise in platelet-activating factor (PAF), which can increase capillary permeability and lead to volume depletion and hypotension, manifesting as acute renal injury via decreased renal perfusion [14]. 

In addition, this study notably found that GAD also is a risk factor for sepsis and increased mortality in patients hospitalized with AP. Severe AP is characterized by a pro-inflammatory state that results in systemic inflammatory response (SIRS) and pancreatic necrosis, followed by an anti-inflammatory response that can result in infection from the translocation of bacteria from a weakened intestinal barrier [16,17]. Similarly, the elevated cytokine response seen in GAD could contribute to the increase of CRP and IL-6, which are pro-inflammatory cytokines that are known to be significant in sepsis [18]. Additionally, the chronic stress that characterizes GAD has been implicated in the downregulation of immune processes, such as T cell and antibody response, which would contribute to an increased infection risk and poor healing [18].

Of interest, GAD in AP patients was found to increase the risk of acute deep vein thrombosis. The pathophysiology likely involves an increase in pro-inflammatory markers, such as TNF-a, IL-1, and IL-6 in chronic stress, which leads to coagulation cascade activation and prevention of fibrinolysis and anticoagulation [19]. Studies have demonstrated that anxiety and stress can lead to a hypercoagulable state, often manifesting as deep venous thrombosis [20,21]. Although studies have found these processes more attributable to acute anxiety states, fibrinogen levels have been found to be elevated in chronic anxiety disorders [21]. In addition, cortisol and corticotropin-releasing factor (CRF), which is released during a chronic stress response, can provoke the formation of thromboses due to endothelial cell dysfunction [20,22]. 

Collectively, the worse outcomes seen in the GAD cohort may be in part related to the patterns of behavior in patients with GAD. Some studies have suggested that patients with GAD have worse medication non-adherence, perhaps due to increased pharmacy costs, decreased access to psychiatrists, adverse effects from medications such as SSRIs and SNRIs, and failed antidepressant trials [23-25]. Financial burden may result not only from medication costs but also from physician billing in self-pay patients, as well as decreased functioning and productivity in the workplace itself, which are common symptoms experienced by GAD patients [24]. An increased frequency of missing outpatient physician appointments may also have a role in the worse outcomes seen in this study [26].

There are some significant limitations to this study. Utilizing patient data from the NIS database is dependent on the ICD codes that were entered by physicians and other medical practitioners. This possible room for error, if codes were under-reported or over-reported, could result in a theoretical distortion of GAD prevalence, or the prevalence of the evaluated outcomes, in patients hospitalized with AP. The validity of the ICD-9 codes cannot be confirmed because of the privacy protection associated with the use of the NIS database that prevents the identification of individual patient data. The medication class that patients utilize for their treatment of GAD also cannot be assessed with the NIS database and could potentially act as cofounders. Finally, while an ICD-9 code for GAD was utilized in this study, there was no ability to factor in the severity of clinical anxiety given the paucity of more specific ICD-9 codes. Despite the limitations in the study design, this research is associated with significant strengths. The most notable strength of this study is its ability to assess patient demographics, hospitalization data, and outcomes at the national level spanning an entire year. In addition, this study was greatly strengthened by the use of a multivariate regression analysis that adjusted for numerous potential confounding factors.

## Conclusions

In patients hospitalized for AP, GAD is an independent risk factor for acute renal injury, sepsis, acute deep vein thrombosis, and inpatient mortality. The increased risk of these conditions could be related to cumulative inflammatory damage that is the result of the coexistence of AP and GAD, which are both individually inflammatory states. Given the increased likelihood of worse outcomes in AP patients with comorbid GAD, it may be beneficial for this specific patient population to have closer monitoring at the time of admission. Hopefully, with closer monitoring of this cohort of patients, early signs of complications may be more quickly intervened upon to reduce the severity of the negative outcomes identified in this study. One possible avenue for further investigation that may be beneficial would be assessing whether the severity of GAD factors into the frequency or severity of the outcomes evaluated in this study. In addition, it would possibly be valuable to study whether other types of chronic stress disorders, such as PTSD or depression, impact the outcomes of AP patients.
